# Sorting of droplets by migration on structured surfaces

**DOI:** 10.3762/bjnano.2.25

**Published:** 2011-04-20

**Authors:** Wilfried Konrad, Anita Roth-Nebelsick

**Affiliations:** 1 University of Tübingen, Institute for Geosciences, Sigwartstrasse 10, D-72076 Tübingen, Germany; 2 State Museum of Natural History Stuttgart, Rosenstein 1, D-70191 Stuttgart, Germany

**Keywords:** microdroplets, microfluidics, surface, surface energy, surface structures

## Abstract

**Background:** Controlled transport of microdroplets is a topic of interest for various applications. It is well known that liquid droplets move towards areas of minimum contact angle if placed on a flat solid surface exhibiting a gradient of contact angle. This effect can be utilised for droplet manipulation. In this contribution we describe how controlled droplet movement can be achieved by a surface pattern consisting of cones and funnels whose length scales are comparable to the droplet diameter.

**Results:** The surface energy of a droplet attached to a cone in a symmetry-preserving way can be smaller than the surface energy of a freely floating droplet. If the value of the contact angle is fixed and lies within a certain interval, then droplets sitting initially on a cone can gain energy by moving to adjacent cones.

**Conclusion:** Surfaces covered with cone-shaped protrusions or cavities may be devised for constructing “band-conveyors” for droplets. In our approach, it is essentially the surface structure which is varied, not the contact angle. It may be speculated that suitably patterned surfaces are also utilised in biological surfaces where a large variety of ornamentations and surface structuring are often observed.

## Introduction

Manipulation of droplets is an issue of great interest in microfluidics. The underlying motivation is the design of microdevices that are able to perform various fluidic processes within dimensions on the micrometer scale [1]. “Lab-on-a-chip” concepts aim at integrating chemical and biochemical processes into chip-like designs that enable the user to carry out tasks in analytical chemistry or bioassay applications [2]. The design of microfluidic batch processes requires a continuous and controlled flow and cycling of suspended droplets of reactants without contamination. Single droplet movement can be achieved with different techniques, such as thermal Marangoni flow, electrowetting and vibration techniques [1]. Specifically designed surfaces can lead to spontaneous droplet movement, even uphill [3].

It is well known [1,4–8] that liquid droplets move towards areas of minimum contact angle if placed on a flat solid exhibiting a gradient of contact angle. Yang et al. [2] devised a hydrophobic micropatterned surface with a gradient in density of the microstructures that lead to droplet movement with maximum speeds of about 60 mm/s. This effect can be obtained by varying 1) chemical contact angle, 2) surface texture, or 3) both parameters.

In this contribution we describe how a controlled droplet movement can be achieved by a surface pattern consisting of cones and funnels whose length scales are comparable to the droplet diameter. In our approach, it is essentially the surface structure which is varied, not the contact angle. The actual movement of the droplet on the continuously varying solid surface pattern depends both on the surface pattern and on the contact angle between droplet and solid. Therefore, it is possible to devise surface patterns which are able to direct droplets differently, depending on their chemical nature.

## Results

First, we derive the surface formation energy of droplets attached to cone shaped protrusions or cavities. Afterwards, we discuss the properties of specific surface patterns composed of cones with varying apex angles.

### Properties of droplets attached to cone shaped protrusions or cavities

**Figure 1:**
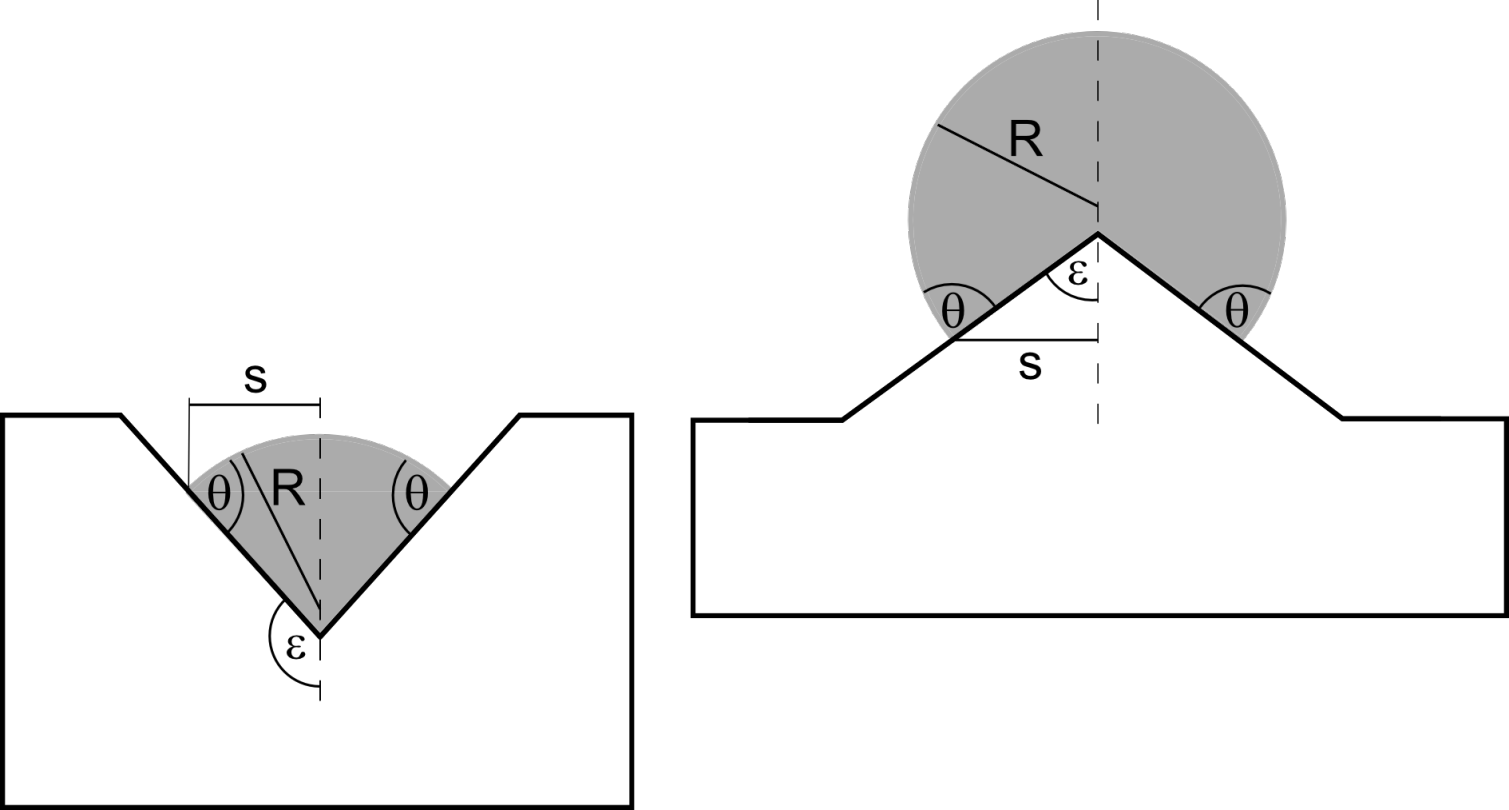
Sphere like droplets of fluid #1 (grey) attached to an axially symmetric solid cone (the broken line represents the symmetry axis). θ: contact angle, *R*: droplet radius, *s*: radius of contact circle (= line where solid, fluid #1 and fluid #2 are in contact), ε: apex half-angle of protrusion (for 0° < ε < 90°, right) or cavity (for 90° < ε < 180°, left).

Consider a sphere-like droplet of radius *R* attached to an axially symmetric, cone shaped protrusion or cavity with apex half-angle ε = 0…180° forming a contact angle θ = 0…180° (see Figure 1). We assume that the droplet is attached in a symmetry preserving way (i.e., the symmetry axes of cone and droplet coincide). We further assume that the droplet consists of a fluid (“fluid #1” in what follows) and is surrounded by a second fluid (“fluid #2” in what follows). One of the fluids must be a liquid, if both fluids are liquids they should be immiscible.

In order to form a droplet in contact with a solid, a surface formation energy *W* has to be provided. If we consider only droplets with constant volume this energy is given by the expression [9]

[1]
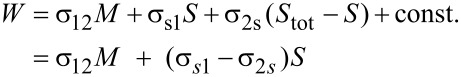


where *S* denotes the attachment area between droplet and solid, *M* is that part of the droplet surface which is in contact with fluid #2 and *S*_tot_ – *S* is the area where plane and fluid #2 are in contact.

σ ≡ σ_12_, σ_s1_ and σ_2s_ denote the surface tensions (or surface energies) with respect to fluid #1/fluid #2, solid/fluid #1 and fluid #2/solid interfaces, respectively. The product σ_2s_*S*_tot_ yields a constant value. Since *W* is defined only up to an arbitrary constant, we can ascribe to it the value –σ_2s_*S*_tot_ which yields the second version of Equation 1. This choice is equivalent to ascribing a vanishing droplet (i.e., *M* → 0, *S* → 0) zero surface energy.

Droplets of constant volume in equilibrium with respect to the surface tensions pulling at them obey the Young Law

[2]
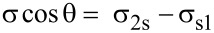


Inserting this relation into Equation 1 we obtain the surface energy *W* of a droplet of fixed volume *V* in equilibrium with respect to surface tensions:

[3]
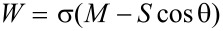


Expressing the surface segments *M* and *S* as well as the contact circle radius *s* and the droplet volume *V* in terms of the quantities *R*, ε and θ, we find from Figure 1

[4]
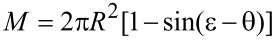


[5]
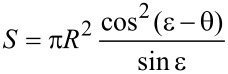


[6]
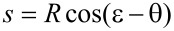


[7]
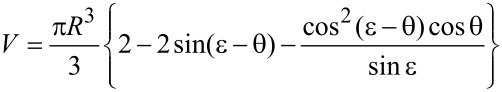


Since the formulas in Equation 4 through Equation 7 encompass cone shaped protrusions and cavities, we designate them both in what follows by the common term “cone”.

Inserting Equation 4 and Equation 5 into Equation 3, we obtain for the surface energy *W* of an equilibrated droplet

[8]
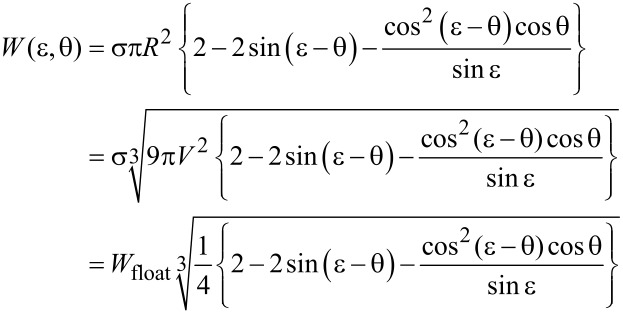


where we have employed equation Equation 7 to replace the droplet radius *R* in favour of the (constant) droplet volume *V* in the second expression.

[9]
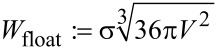


denotes the surface formation energy of a spherical droplet of volume *V* which consists of fluid #1 and floats (i.e., without contact to the solid) within fluid #2.

**Figure 2:**
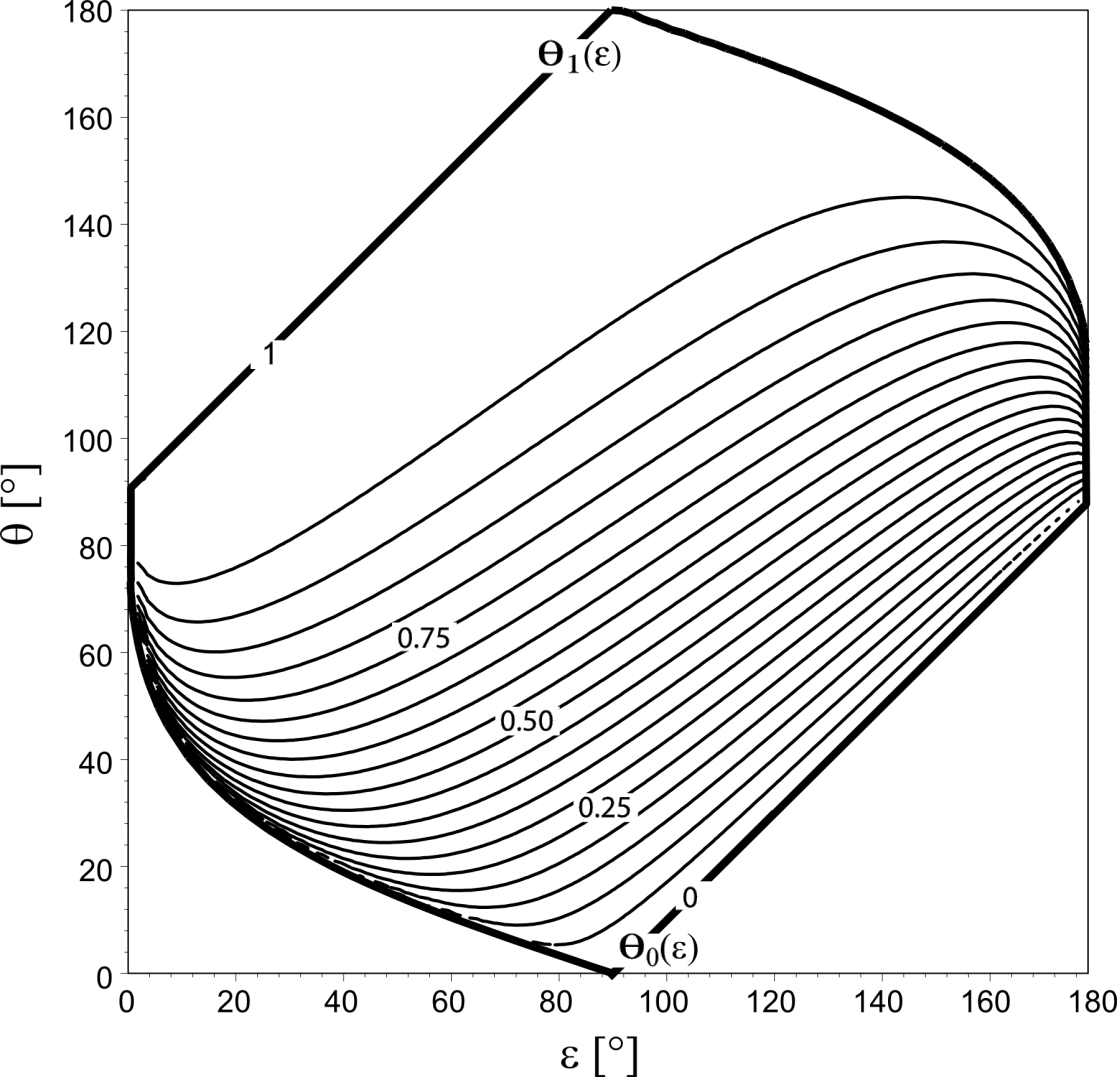
Thin lines: Curves of constant surface formation energy *W* (ε, θ) of an equilibrated droplet of volume *V*, according to Equation 8. Values of *W* are given as multiples of the surface energy *W*_float_ of an unattached spherical droplet (see Equation 9). The lines represent the values *W*/*W*_float_ = 0.00, 0.05, 0.10,…0.95, 1.00, starting from the lowermost line. Thick lines: the functions θ = Θ_0_(ε) and θ = Θ_1_(ε) (defined in Equation 11 and Equation 12, resp.) envelop the (ε, θ)-pairs related to equilibrated droplets of volume *V* attached to a cone. The region below θ = Θ_0_(ε) has to be excluded because combinations of apex half-angle ε and contact angle θ in this region lead to *V* < 0. The curve θ = Θ_1_(ε) indicates droplets with the same surface energy as freely floating droplets without contact to the solid.

A closer look at the terms in the braces of expression Equation 7 reveals that certain (ε, θ)-combinations have to be excluded because they represent (non-physical) negative droplet volumes. The admissible (ε, θ)-ranges (equivalent to droplets with *V* ≥ 0) are given by (see also Figure 2):

[10]
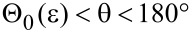


where (see Equation 11)

[11]
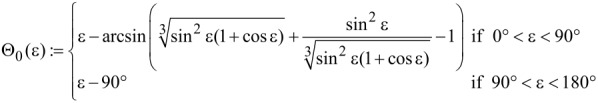


The function Θ_0_(ε) is calculated by setting *V* = 0 in Equation 7. Comparison of Equation 7 and Equation 8 shows the equivalence of the conditions *V* ≥ 0 and *W* (ε, θ) ≥ 0.

Below, the question will arise whether a freely floating droplet of surface energy *W*_float_ gains energy if it attaches to a cone defined by a given (ε, θ)-pair or whether this process consumes energy. The answer is found by equating the expressions in Equation 8 and Equation 9: Solving for θ, one obtains a curve

[13]
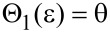


with (see Equation 12)

[12]
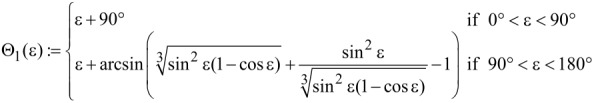


The curve Θ_1_(ε) = θ (Figure 2) divides the (ε, θ)-plane into two regions: Cones that are generated by (ε, θ)-pairs below it imply *W*(ε, θ) < W_float_, that is, a freely floating droplet of surface energy *W*_float_ gains surface energy if it chooses to attach to such a cone. Cones characterised by (ε, θ)-pairs above Θ_1_(ε) > θ require for attachment the energy *W* (ε, θ) > *W*_float_, i.e., attachment of a freely floating droplet would consume energy.

If ε is held constant, *W* (ε, θ) is a continuous and increasing function of θ, i.e., θ_2_ ≥ θ_1_ implies *W* (ε, θ_2_) ≥ *W* (ε, θ_1_). This can be seen by calculating the slope of Equation 8 with respect to θ which is positive for ε, θ = 0…180°.

**Figure 3:**
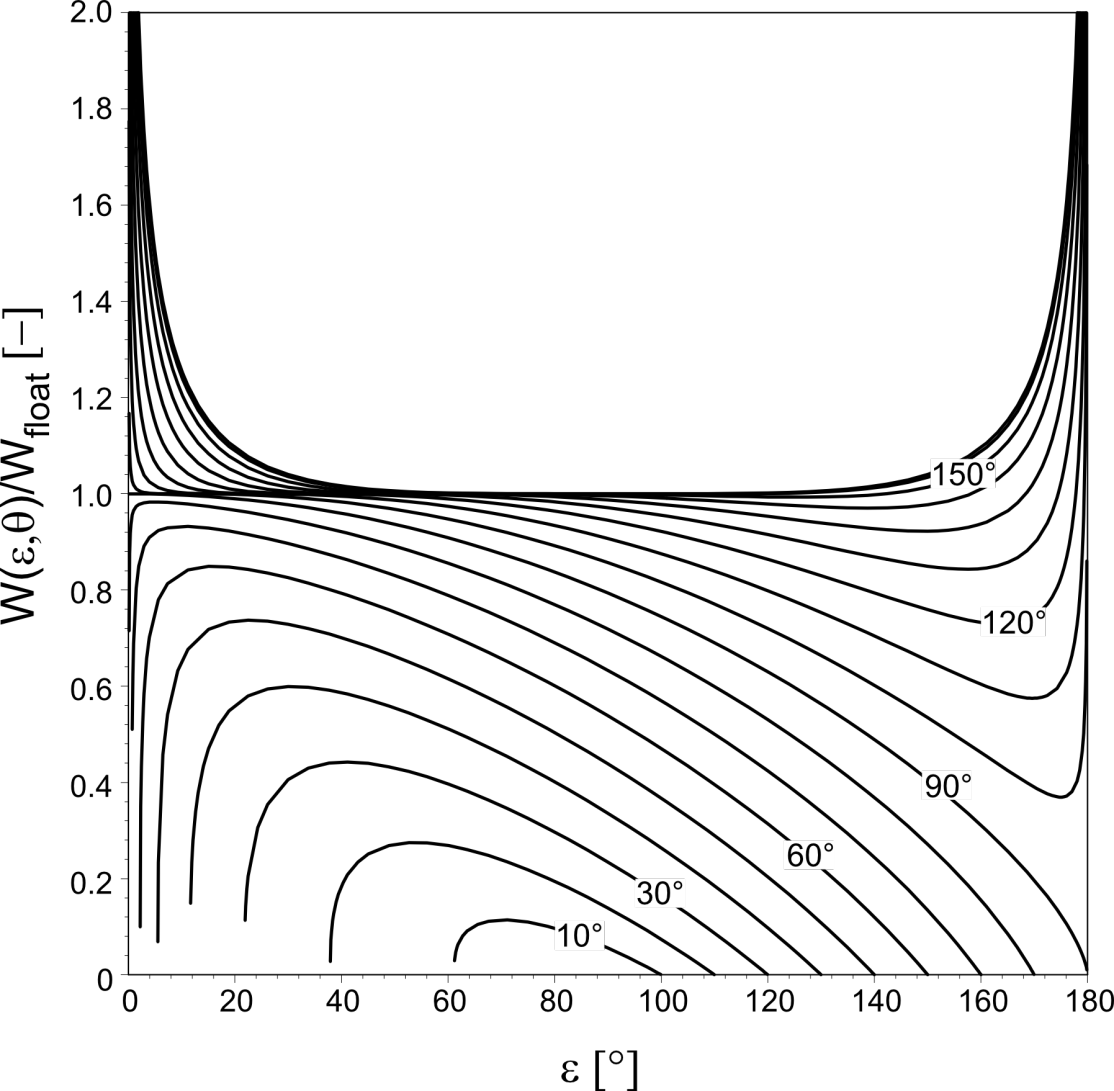
Surface formation energy *W* (ε, θ) of an equilibrated droplet of volume *V*. Values of *W* are given as multiples of the surface energy *W*_float_ = 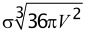 of a floating spherical droplet of volume *V*. The curves are distinguished by the value of the contact angle, namely θ = 10°, 20°, 30°…170°. Curves related to 0° < θ < 90° show *maxima* which lie all within the range 0° < ε < 90°, whereas curves with 90° < θ < 180° show *minima* all of which are located within the range 90° < ε < 180°.

Figure 3 illustrates the behaviour of *W* (ε, θ) if θ is kept constant: *W* (ε, θ) exhibits extrema with respect to ε whose positions depend on the value of θ. For 0° ≤ θ ≤ 90°, the curves *W* (ε, θ = const.) show *maxima* within the range ε = 0°…90°, more precisely

[14]
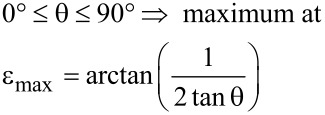


whereas the curves with 90° ≤ θ ≤ 180° have *minima* within a range ε = 90°…180°, i.e.,

[15]
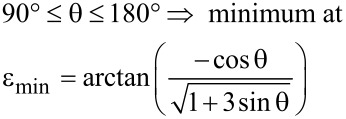


Curves with 90° ≤ θ ≤ 180° have also saddle points at ε_saddle_ = θ – 90°. For ε → 90° both protrusions and cavities degenerate to flat surfaces.

### Hypothetical applications

The main results of the previous section are as follows (see also Figure 2 and Figure 3):

1. The surface energy *W* (ε, θ) of a droplet attached to a cone of apex half-angle ε in a symmetry-preserving way is smaller than the surface energy *W*_float_ of a freely floating droplet, provided that the (ε, θ)-pair lies between the curves Θ_0_(ε) = θ and Θ_1_(ε) = θ (Figure 2).

2. If the value of the contact angle is fixed and lies within the interval 0° < θ < 90°, the surface energies of droplets sitting on cones whose apex half-angle ε are close to the value ε_max_ given in Equation 14 are higher than the surface energies of droplets attached to cones with greater or smaller apex half-angles.

3. If the value of the contact angle is fixed and lies within the interval 90° < θ < 180°, *W* (ε, θ) exhibits a minimum at ε = ε_min_ (Equation 15). Thus, the surface energies of droplets sitting on cones which are very differently shaped (in terms of apex half-angle ε) are higher than the surface energies of droplets attached to cones whose shape is more similar to ε_min_.

**Figure 4:**
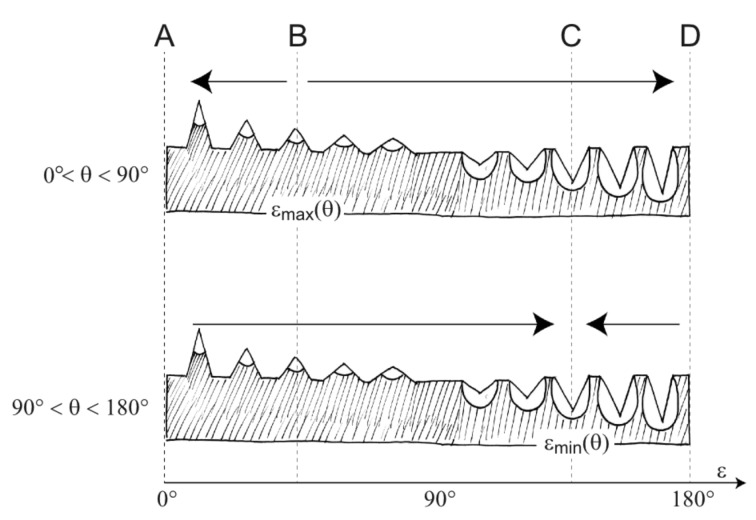
Line-up of cones. The apex half-angle ε increases from left to right. For θ = const. and 0° < θ < 90° (upper part), the surface energy *W* (ε, θ) has a maximum at point B and for 90° < θ < 180° (lower part) a minimum at point C. The arrows indicate directions of decreasing surface energy. In section B–C they point into the same, in sections A–B and C–D, however, into opposite directions (drawing: Birgit Binder, Tübingen).

The features just discussed permit speculation about constructing “band-conveyors” for droplets. Such a “band-conveyor”, capable of “passing down” droplets from cone to cone, might be generated by arranging cones with increasing values of ε (but fixed θ) in one- or two-dimensional patterns. Figure 4 illustrates the basic idea: Upper and lower part of the figure show the same line-up of cones. The apex half-angle ε increases from left to right. If the fixed contact angle lies within 0° < θ < 90° (upper part of Figure 4), the function *W* (ε, θ) has a maximum at point B (i.e., ε_max_(θ)). Thus, the energy difference Δ*W* := *W*_float_ – *W* (ε, θ) which is required to detach a droplet from a cone has its minimum at point B. With increasing distance from B, droplets are increasingly stronger bound to their substrate, that is, Δ*W* increases towards A and D. If the fixed contact angle lies between 90° < θ < 180° (lower part of Figure 4), the minimum of *W* (ε, θ) is located at point C (i.e., ε_min_(θ)). Hence, Δ*W* increases if point C is approached from A or D.

If both lyophilic (i.e., 0° < θ < 90°) and lyophobic droplets (i.e., 90° < θ < 180°) reside on the landscape of cones of Figure 4, it appears that both droplet species experience an increase of Δ*W* from point B towards C. In sections A–B and C–D, however, the variation of Δ*W* points into opposite directions for the two droplet species.

**Figure 5:**
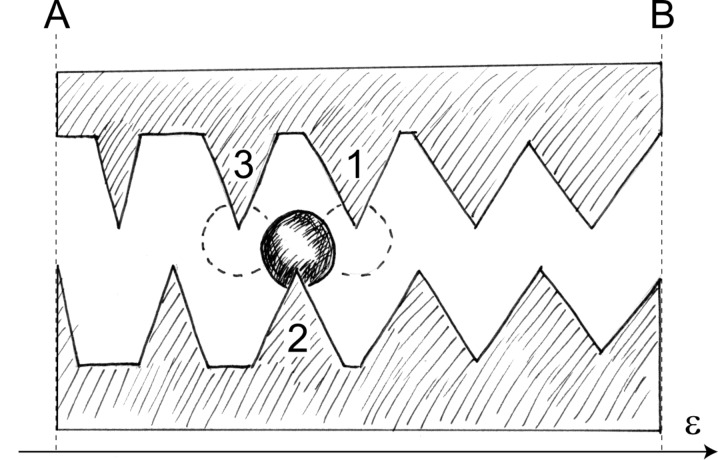
Double line-up of cones similar to a zip fastener constructed from the left part of Figure 4. The apex half-angle ε of the cones increases from point A (ε ≈ 0°) to point B (ε = ε_max_). Apart from one or two cones, the droplet does not touch the solid substrate (drawing: Birgit Binder, Tübingen).

Perhaps, these findings can be utilised to construct a “band-conveyor” for droplets. We present two ideas how this might be achieved:

1. Consider Figure 5. The double line-up of cones similar to a zip fastener is constructed from the left part of Figure 4. The apex half-angle ε of the cones increases from point A (ε ≈ 0°) to point B (ε = ε_max_). According to Equation 14, a lyophilic droplet attached to cone #2 is in a lower state of surface energy than a droplet at cone #1, but in a higher energetic state than the droplet at cone #3. If the dimensions of droplet and cones, the contact angle between them and the temperature of the arrangement are suitably chosen, thermal oscillations of the droplet around its position of symmetry at cone #2 may bring it in contact with cone #1 or cone #3. Due to the gradient of *W* (ε, θ) with respect to ε, for a lyophilic droplet it is energetically attractive to move to cone #3 (towards lower values of *W* (ε, θ)), but not to cone #1. Thus, lyophilic droplets should finally get to point A. For lyophobic droplets, a similar reasoning applies, which starts, however, from Equation 15 instead of Equation 14. Hence, lyophobic droplets should migrate towards point B.

2. If the cones are farther apart than in Figure 5 (more like in Figure 4), the droplets have to detach completely from a cone before they come in contact with the next one. Similarly as above, if droplet and cone dimensions, contact angle and temperature are favourable, the interaction of the droplet with the thermally agitated particles constituting fluid #2 may outweigh the binding energy Δ*W* = *W*_float_ – *W* (ε, θ) between droplet and solid substrate. Since the thermal agitation fluctuates randomly, the transfer of low amounts of energy onto the droplet occurs more frequently than the transfer of high amounts of energy, on an average. Consequently, the mean residence time of a droplet sitting on a cone increases with increasing binding energy Δ*W*. Therefore, lyophilic droplets attached to cones close to point B (see upper part of Figure 4) leave the cones more often than droplets farther away from B. Doing so, a droplet may jump – with equal probability – either to the left or to the right: In case its next attachment is closer to B, its residence time is shorter than before, if it moves to a cone farther away from B, it will remain there longer, on an average. The overall effect of this is a (net) movement of lyophilic droplets towards B.

Lyophobic droplets behave similarly, they move also in the direction of decreasing binding energy Δ*W*. This means, in terms of the lower part of Figure 4, that they move away from point C, towards points A and D.

Up to now, we have simply assumed that detached droplets re-attach to the tips of the cones. Of course, this cannot be taken for granted. One may exclude this eventuality by assembling the landscape of cones of Figure 4 from two materials with different contact angles: (a) bulk material with a very high contact angle (dotted areas in Figure 4), implying a very small Δ*W*, and (b) employing material with smaller contact angle for the cones (white areas in Figure 4). Alternatively, one might apply hair- or pillar-like structures which are smaller than the cones by an order of magnitude or so to the dotted areas in Figure 4. Droplets coming in contact with these structures should experience a Cassie state, leading also to very high effective contact angles [8].

Notice that the mechanisms depicted in Figure 4 and Figure 5 predict droplet migration in opposite directions. Concentrating on Figure 4, a possible application of the suggested mechanism arises, for example, from exploiting only parts of Figure 4: Arranging the landscape of cones in a twodimensional, radial pattern such that point A is close to the centre and B represents the outer fringe of a circular disc, lyophilic droplets would migrate towards the centre whereas lyophobic droplets would migrate away from it. A reverse migration order should result if the roles of A and B are interchanged or if point C is chosen as the centre and point D as the periphery.

## Discussion

The specific surface patterning is not only able to initiate a spontaneous and directed movement of droplets. According to the actual surface energy, droplets with different chemical content will move to different directions. This effect thus enables not only droplet transport but also droplet sorting. According to the surface pattern, different motion patterns and behaviour can be achieved. For example, different chemical reactants can be directed to different “assembly” lines. Also the speed of the droplets can be controlled.

Surfaces similar to our patterns are not uncommon in nature. Insects show a wide variety of ornamentations of their cuticle, their compound eyes and wings [10]. Plant surfaces are also known to develop a huge variety of patterns on different length scales [11]. A prominent example are the leaf wax structures leading to superhydrophobicity and the Lotus-effect [12]. Larger structures are also common, e.g., trichomes (leaf hairs) or wart-like structures. Stomata, the micropores for gas exchange on leaves, are often particularly decorated. The stomata of *Equisetum* (horse-tail), for example, show wart-like protuberances around the central slit, while the stomata of *Gingko* are surrounded by cone-shaped structures. It might be speculated that directed transport of tiny droplets may also be involved in these cases: Stomata have to be protected from being covered by a water film, and therefore the development of water-repellent structures should be beneficial. This applies not only to large drops, as are delivered by rain precipitation, but also to droplets in the μm-range which are deposited during fog or mist events. Specifically shaped surface patterns may be able to repel not only large water drops but also direct microdroplets according to specific strategies.
